# Metasurface-integrated elliptically polarized laser-pumped SERF magnetometers

**DOI:** 10.1038/s41378-024-00715-3

**Published:** 2024-07-19

**Authors:** Zihua Liang, Jinsheng Hu, Peng Zhou, Lu Liu, Gen Hu, Ankang Wang, Mao Ye

**Affiliations:** 1 School of Instrumentation and Optoelectronic Engineering, Beihang University, Beijing, 100191 China; 2 Institute of Large-scale Scientific Facility and Centre for Zero Magnetic Field Science, Beihang University, Beijing, 100191 China; 3 Hangzhou Institute of Extremely-Weak Magnetic Field Major National Science and Technology Infrastructure, Hangzhou, Beihang Hangzhou Innovation Institute, Hangzhou, 310051 China

**Keywords:** Optical materials and structures, Nanoscale devices

## Abstract

The emergence of biomagnetism imaging has led to the development of ultrasensitive and compact spin-exchange relaxation-free (SERF) atomic magnetometers that promise high-resolution magnetocardiography (MCG) and magnetoencephalography (MEG). However, conventional optical components are not compatible with nanofabrication processes that enable the integration of atomic magnetometers on chips, especially for elliptically polarized laser-pumped SERF magnetometers with bulky optical systems. In this study, an elliptical-polarization pumping beam (at 795 nm) is achieved through a single-piece metasurface, which results in an SERF magnetometer with a high sensitivity reaching 10.61 fT/Hz^1/2^ by utilizing a ^87^Rb vapor cell with a 3 mm inner diameter. To achieve the optimum theoretical polarization, our design combines a computer-assisted optimization algorithm with an emerging metasurface design process. The metasurface is fabricated with 550 nm thick silicon-rich silicon nitride on a 2 × 2 *cm*^2^ SiO_2_ substrate and features a 22.17° ellipticity angle (a deviation from the target polarization of less than 2%) and more than 80% transmittance. This study provides a feasible approach for on-chip polarization control of future all-integrated atomic magnetometers, which will further pave the way for high-resolution biomagnetism imaging and portable atomic sensing applications.

## Introduction

In recent years, miniaturized atomic magnetometers based on the self-exchange relaxation-free (SERF) principle^1^ have been continuously broadening our horizon in biomagnetism imaging research because of their extremely high sensitivity and remarkable flexibility, and they have exciting potential for the diagnosis of heart and brain disease^2–5^. To improve the resolution of biomagnetism imaging, integrated atomic magnetometers that utilize nanofabrication technology to significantly reduce the volume and increase the number of channels have become research hotspots^6–9^. Among the numerous components of atomic magnetometers, bulky optical components have become the major obstacle for reducing volume compared with already integrated electromagnetic circuits.

The accuracy of the polarization conversion will directly affect the component of the photon spin angular momentum carried by the laser along the direction of the pumping light, thereby affecting the pumping efficiency and polarizability and ultimately affecting the scale coefficient and signal-to-noise ratio of the magnetometer. Therefore, precise transformation of the optical polarization is vital for increasing the sensitivity of atomic magnetometers^10^. However, conventional bulky polarization-transformation systems require adjusting the angle between the fast axis of the half-wave plate and the quarter-wave plate to transform incoming linearly polarized light into the desired polarization state^11,12^, which is challenging to integrate for all-integrated atomic magnetometers. Integrated photonic devices^8,13–15^, exemplified by metasurfaces, have shown the capability of versatile polarization control with wafer-lever integration, enabling ultracompact packaging of optical devices into miniaturized atomic magnetometers. Moreover, the design of chip-scale nanophotonic components can be enhanced by emerging computer-assisted algorithms^16^, which further facilitate the optimization of the performance of atomic sensors.

To immunize our integrated atomic magnetometer system against vertical-cavity surface-emitting laser (VCSEL)^17^ laser intensity noise, an elliptically polarized light pumping scheme is utilized to build our magnetometer system in this study. An elliptical-polarization pumping beam at 795 nm is transformed by a single-piece metasurface, leading to an SERF magnetometer with a high sensitivity reaching 10.61 fT/Hz^1/2^. This result is comparable to those of the current state-of-the-art SERF magnetometers^11,12,18,19^, which utilize traditional bulky optical components. An elliptically polarized beam is designed to achieve the theoretically optimum polarization state by combining the metasurface polarization transform method with a computer-assisted optimization algorithm. The device is made with a 550 nm-thick silicon-rich silicon nitride layer on a SiO_2_ substrate, and it features an ellipticity angle of 22.17 degrees (less than 2% deviation from the target 22.5 degree polarization angle) and a transmittance of greater than 80%. The aim of this study is to demonstrate that polarization-transformation metasurfaces relying on nanofabrication technology can entirely replace traditional bulky optical devices in atomic magnetometers, which has substantial potential for achieving exponential volume reduction and resolution improvement. Furthermore, our method paves the way for the development of all-integrated atomic magnetometers, which are promising for future wearable atomic devices and high-spatial-resolution biomagnetism imaging.

## Results & Discussion

### Approach to designing an integrated elliptically polarized SERF magnetometer

Currently, there are two predominant detection schemes for SERF magnetometers, which involve monitoring either the intensity or polarization of transmitted light. The first detection scheme measures the magnetic field based on the intensity variations of a single circularly polarized light. Although characterized by a simple structure, the performance of this scheme is significantly impacted by fluctuations in light intensity^20^. Consequently, the majority of compact magnetometer systems connect the sensor to the laser source via optical fibers^4,21^ rather than directly integrating a compact VCSEL laser with fluctuating intensity into the magnetometer, which increases the complexity and decreases the robustness of the magnetometer system. The second detection scheme, the optical polarization rotation detection method^22^, detects the magnetic field by measuring the magneto-optical rotation angle of linearly polarized light due to the circular birefringence of the vapor cell. Although this scheme effectively reduces the impact of fluctuating light intensity through differential detection techniques, its complex structure still presents a challenge for the further miniaturization of magnetometers. Therefore, there are still many problems to be solved in the application of the above two schemes in all-integrated magnetometers.

**Fig. 1:**
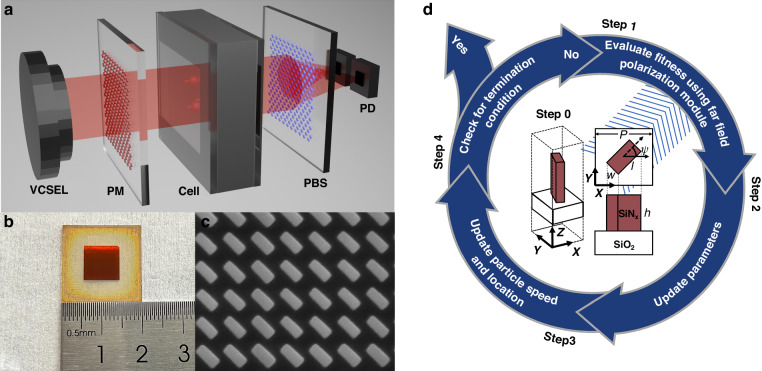
Concept sketch, design and SEM image of polarization-transformation metasurface. **a** Conceptual sketch of the integrated elliptically polarized laser-pumped SERF magnetometer. PM polarization-transformation metasurface, PBS polarization beam splitter, PD photodiode. **b** Photograph of the fabricated polarization-transformation metasurface. **c** Scanning electron microscopy (SEM) image of the polarization-transformation metasurface. **d** Algorithm flow chart for designing the polarization-transformation metasurface

In addition to the previously mentioned schemes, integrated elliptically polarized light magnetometers, for which a conceptual sketch is shown in Fig. 1a, have enhanced the focus of research on all-integrated atomic magnetometers^11,23^ because of their straightforward optical path design and remarkable ability to reduce fluctuation noise in VCSEL light sources^17,24^. In this scheme, circularly polarized component in elliptically polarized light is utilized to pump alkali atoms^11^, and linearly polarized component is utilized to detect changes in atom spin polarization through planar integrated polarization beam splitters^7,8,13^. The ellipticity of the beam after the polarization-transformation device can be described by $elli=\frac{b}{a}=\,\tan \varepsilon$, where *a* and *b* are the lengths of the principal semiaxes and $\varepsilon$ is the ellipticity angle^25^.

After passing through the polarization-transformation device, the elliptically polarized beam with an angle *ϕ* to the incident linearly polarized light can be represented by the Jones matrix as

1 $${E}_{1}=\left[\begin{array}{c}\cos \varepsilon \,\cos \phi +i\,\sin \varepsilon \,\sin \phi \\ \cos \varepsilon \,\sin \phi -i\,\sin \varepsilon \,\cos \phi \end{array}\right]$$

In terms of a circular polarization basis,

2 $$\begin{array}{l}{E}_{1}={E}_{L}+{E}_{R}=(\cos \varepsilon -\,\sin \varepsilon )\left[\begin{array}{c}1\\ i\end{array}\right]{e}^{-i\phi }\\\qquad\qquad\qquad\qquad+\,(\cos \varepsilon +\,\sin \varepsilon )\left[\begin{array}{c}1\\ -i\end{array}\right]{e}^{i\phi}\end{array}$$

The propagation of light through a Rb atomic vapor cell of length $z$ is

3 $${E}_{2}=(\cos \varepsilon -\,\sin \varepsilon )\left[\begin{array}{c}1\\ i\end{array}\right]{e}^{-i\phi }{e}^{i{k}_{L}z}+(\cos \varepsilon +\,\sin \varepsilon )\left[\begin{array}{c}1\\ -i\end{array}\right]{e}^{i\phi }{e}^{i{k}_{R}z}$$

In the atomic vapor cell, the two circular components of light experience different indices of refraction ${k}_{R}=2\pi v{n}_{R}/c$ and ${k}_{L}=2\pi v{n}_{L}/c$ under the influence of a weak magnetic field^10,11^.

4 $${n}_{R}=1+\kappa (1+{P}_{z})L(v)/v$$

5 $${n}_{L}=1+\kappa (1-{P}_{z})L(v)/v$$

Here, $\kappa =n{r}_{e}{c}^{2}f/4\pi$, where *n* is the alkali-metal density, *r*_*e*_ is the classical electron radius, and *f* = 1/3 is the oscillator strength for the *D*_1_ transition; *P*_*z*_ is the electron-spin polarization; *v* is the laser frequency; and $L(v)=\frac{(v-{v}_{0})\,+\,i({\varGamma }_{{\rm{D}}1}/2)}{{(v-{v}_{0})}^{2}\,+\,{({\varGamma }_{{\rm{D}}1}/2)}^{2}}$ is a complex Lorentzian profile with its full width at half maximum (FWHM) ${\varGamma }_{{\rm{D}}1}$ centered at *v*_0_.

6 $${E}_{2}={e}^{i({k}_{L}+{k}_{R})z/2}\left[\begin{array}{c}\cos \varepsilon \,\cos (\phi +\theta )+i\,\sin \varepsilon \,\sin (\phi +\theta )\\ \cos \varepsilon \,\sin (\phi +\theta )-i\,\sin \varepsilon \,\cos (\phi +\theta )\end{array}\right]$$

where $\theta =({k}_{R}-{k}_{L})z/2=\frac{\pi }{\lambda }({n}_{R}-{n}_{L})z=\frac{n{r}_{e}cf\mathrm{Re}[L(v)]}{2}{P}_{z}z$ represents the optical rotation angle.

7 $${E}_{2}={e}^{i({k}_{L}+{k}_{R})z/2}\left[\begin{array}{c}(a\,\cos \phi +ib\,\sin \phi )\cos \theta -(a\,\sin \phi -ib\,\cos \phi )\sin \theta \\ (a\,\sin \phi -ib\,\cos \phi )\cos \theta +(a\,\cos \phi +ib\,\sin \phi )\sin \theta \end{array}\right]$$

The equation above expresses the transformation of elliptically polarized light after passing through the polarization transform device. Moreover, the vapor cell induces a rotation angle *θ* in the elliptically polarized light, which can be detected by an integrated polarization beam splitter^8,13^ and photodetectors.

The sum *S* and difference *D* of the signals in the two photodetectors are given by

8 $$S={e}^{i({k}_{L}+{k}_{R})z}(1+i\,\sin 2\varepsilon \,\sin 2(\phi +\theta ))$$

9 $$D={e}^{i({k}_{L}+{k}_{R})z}\cos 2\varepsilon \,\cos 2(\phi +\theta )$$

To facilitate subsequent differential measurements, the inclination angle of the initial elliptically polarized light (*ϕ*) is generally set to $\frac{\pi }{4}$. For $\phi =\frac{\pi }{4}$, the difference in the photodiode signal is

10 $$D={e}^{i({k}_{L}+{k}_{R})z}\cos 2\varepsilon \,\sin 2\theta$$

The spin polarization of the alkali-metal atoms in the SERF regime can be described by the Bloch equation^10,26,27^:

11 $$\frac{d{\bf{P}}}{dt}=\gamma {\boldsymbol{B}}\times {\bf{P}}+\frac{1}{q}[{R}_{op}(s\hat{z}-{\bf{P}})-{R}_{rel}{\bf{P}}]$$

where **B** is the external magnetic field, *γ* is the gyromagnetic ratio of the atomic spin, *q* is the nuclear slowing-down factor, *R*_*op*_ is the pumping ratio, and *R*_*rel*_ is the relation ratio. Generally, the light polarization of the photon spin component along the pumping direction is expressed as $s=\overrightarrow{s}\cdot \hat{z}$; *s*ranges from −1 to +1, where *s* = −1 corresponds to ${\sigma }^{-}$ light, *s* = 0 corresponds to linearly polarized *π* light, and *s* = + 1 corresponds to ${\sigma }^{+}$ light. The average photon spin *s* is given by

12 $$s=-\,\sin 2\varepsilon$$

If the magnetic field changes slowly $(\frac{d{\bf{P}}}{dt}=0)$ and ${B}_{y}={B}_{z}=0$,

13 $${P}_{z}=\frac{s{R}_{op}({R}_{op}+{R}_{rel})}{{({R}_{op}+{R}_{rel})}^{2}+{\gamma }^{2}{B}_{x}^{2}}$$

The difference in the photodiode signal is

14 $$D\propto \,\cos 2\varepsilon \,\sin (\sin 2\varepsilon )\propto \,\sin 4\varepsilon$$

The ellipticity angle *ε* determines the intensity of the pumping light (circularly polarized) and the probe light (linearly polarized), which affects the polarizability of the alkali vapor cell and the amplitude of the final response signal. To improve the amplitude of the response signal and the signal-to-noise ratio, the response of the differential signal should be increased as much as possible. Therefore, when $\sin 4\varepsilon =1$, $\varepsilon =\pi /{{8=22.5}^{\circ}}$ represents the maximum value of the output signal, that is, the optimal value of the ellipticity. In conclusion, the optimal optical ellipticity is $\varepsilon ={{22.5}^{\circ}}$ for a larger scale factor in an elliptically polarized SERF magnetometer, and the transformation precision of the polarization device substantially impacts the magnetometer’s performance.

In this study, an algorithm that we developed based on the particle swarm optimization (PSO) algorithm is implemented in MATLAB and combined with three-dimensional finite-difference time-domain (FDTD) simulation to obtain an effective polarization-transforming metasurface. This polarization-transformation metasurface is constructed by employing a standard glass wafer with a 550 nm thick layer of silicon-rich silicon nitride and consists of miniaturized blocks whose size is determined by the optimization algorithm. The target ellipticity for the optimization was defined to maximize the amplitude of the differential signal of the photodiode ($\varepsilon ={{22.5}^{\circ}}$). A photograph of the polarization-transformation metasurface and a scanning electron microscope image of the fabricated device are presented in Fig. 1b, c, respectively. To facilitate experimental verification, a 9 × 9 *mm*^2^ SiNx structure is prepared on a 2 × 2 *cm*^2^ SiO_2_ substrate as a polarization transforming device for testing.

### Integrated polarization-transformation metasurface

To precisely transform the polarization, the propagation phase method^28,29^ and geometric phase method^30,31^ are combined to transmit incident linearly polarized light into light with the desired polarization, which is described in the Jones matrix. As shown by the red block in Fig. 1d, the nanopillar represents the fundamental building unit of a polarization-transformation metasurface. Considering the propagation phase, the phases and amplitudes of the two mutually perpendicular polarization components passing through the nanopillar are expressed by the Jones matrix as

15 $${E}_{out}=\left[\begin{array}{cc}k{e}^{-i\frac{{\varGamma }}{2}} & 0\\ 0 & {e}^{i\frac{{\varGamma }}{2}}\end{array}\right]{E}_{in},$$

where ${\varGamma }$ represents the phase difference. As shown in the equation, after light passes through the nanopillar along the z direction, the phase difference between the two polarization components is ${\Gamma }$, and the ratio of the transmittance is *k*. On the basis of the propagation phase, the nanopillar rotates along the z-axis while maintaining the incident polarization direction, which can be expressed as follows:

16 $$\begin{array}{c}T=R(-\psi )\left[\begin{array}{cc}k{e}^{-i\frac{{\varGamma }}{2}} & 0\\ 0 & {e}^{i\frac{{\varGamma }}{2}}\end{array}\right]R(\psi )\\ =\left[\begin{array}{cc}\cos \psi & -\sin \psi \\ \sin \psi & \cos \psi \end{array}\right]\left[\begin{array}{cc}k{e}^{-i\frac{{\varGamma }}{2}} & 0\\ 0 & {e}^{i\frac{{\varGamma }}{2}}\end{array}\right]\left[\begin{array}{cc}\cos \psi & \sin \psi \\ -\sin \psi & \cos \psi \end{array}\right]\\ =\left[\begin{array}{cc}k{e}^{-i\frac{{\varGamma }}{2}}{\cos }^{2}\psi +{e}^{i\frac{{\varGamma }}{2}}{\sin }^{2}\psi & (k{e}^{-i\frac{{\varGamma }}{2}}-{e}^{i\frac{{\varGamma }}{2}})\sin \psi \,\cos \psi \\ (k{e}^{-i\frac{{\varGamma }}{2}}-{e}^{i\frac{{\varGamma }}{2}})\sin \psi \,\cos \psi & k{e}^{-i\frac{{\varGamma }}{2}}{\sin }^{2}\psi +{e}^{i\frac{{\varGamma }}{2}}{\cos }^{2}\psi \end{array}\right]\end{array}$$

where $R(\psi )$ denotes the rotation matrix for an angle $\psi$ in the counterclockwise direction. The x-direction linearly polarized light incident to the structure is considered. The transmitted light can be expressed as

17 $${E}_{out}=T\left[\begin{array}{c}1\\ 0\end{array}\right]=\frac{1}{2}\left(\left(k{e}^{-i\frac{{\Gamma }}{2}}-{e}^{i\frac{{\Gamma }}{2}}\right)\left[\begin{array}{c}\cos 2\psi \\ \sin 2\psi \end{array}\right]+\left(k{e}^{-i\frac{{\Gamma }}{2}}+{e}^{i\frac{{\Gamma }}{2}}\right)\left[\begin{array}{c}1\\ 0\end{array}\right]\right)$$

This equation represents that the transmitted light contains not only the component in the polarization direction of the incident light but also the polarization component in the direction of twice the rotation angle of the nanopillar. With a 45-degree fixed deflection angle, two mutually perpendicular polarization components constitute a new polarization state distinct from the incident light, and any transmitted polarization state can be constructed by changing the phase difference ${\Gamma }$ and transmittance ratio $k$ of these two polarization components. The ellipticity of the outgoing beam $|elli|$ can be determined from the amplitude ratio and phase difference:

18 $${E}_{x}=k\,\cos (\varphi +\Gamma ),{E}_{y}=\,\cos (\varphi )$$

19 $$|E|=\sqrt{{E}_{x}^{2}+{E}_{y}^{2}}$$

20 $$|elli|=\frac{b}{a}=\frac{\min |E|}{\max |E|}$$

**Fig. 2:**
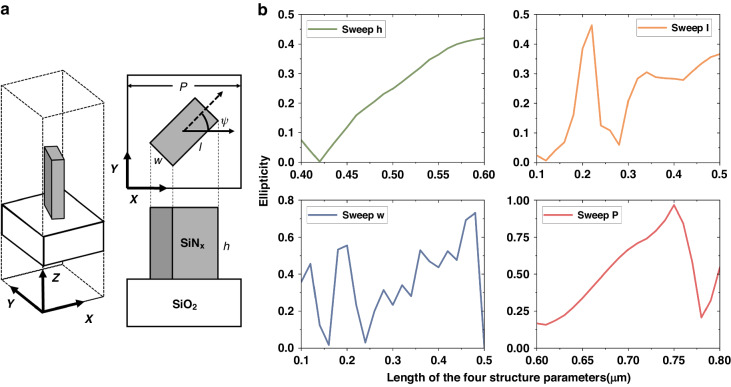
Schematic diagram of nanopillar and the influence of structural parameters on polarization-transformation. **a** Conceptual sketch of a nanopillar. **b** One-dimensional sweep for each structural parameter

The polarization state of the transmitted light is affected by multiple structural parameters, including the period (P), length (l), width (w) and height (h) of the nanopillars, as shown in Fig. 2a.

**Fig. 3:**
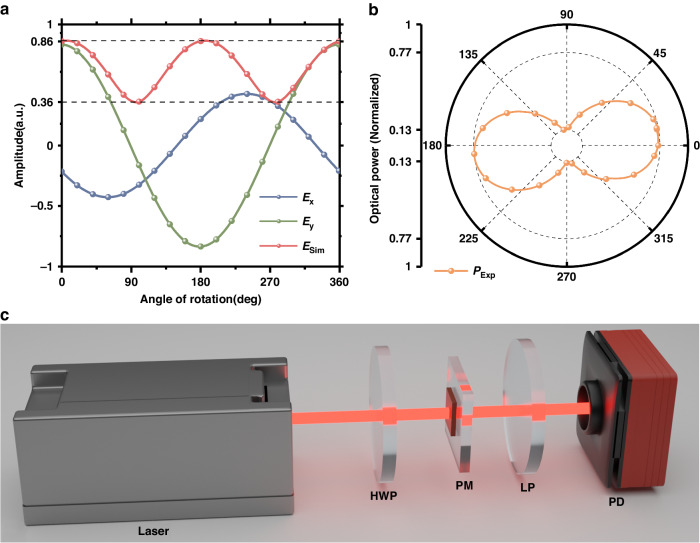
Simulation results and experimental tests for the polarization-transformation metasurface. **a** Simulation results for the polarization-transformation metasurface. The red line illustrates the amplitude of the transmitted beam at various angles, while the blue and green lines correspond to the x and y polarization components of the beam amplitude, respectively. **b** Experimental results for the optical power. **c** Schematic diagram of the testing process for the polarization-transformation metasurface

As shown in Fig. 2b, each structural parameter of the nanopillars is scanned in one dimension to demonstrate its effect on the degree of polarization for transmitted light. To achieve the closest phase difference and amplitude ratio between the two polarization components, traditional parametric scanning schemes must scan these structural parameters over a large range with very small scanning intervals. After that, structural parameters satisfying the specific phase difference and amplitude ratio must be identified from this large database to achieve a specific polarization of the transmitted light. However, for this type of optimization problem, the reverse optimization algorithm has higher design efficiency and accuracy^32–34^. The PSO algorithm is employed for direct inverse parameter selection in this study, utilizing the polarization state of the transmitted beam as the fitness function. A flowchart of the algorithm is shown in Fig. 1d, and the process is described in detail in the Materials and Methods section. Only eight iterations are needed to meet the target ellipticity requirements and jump out of the loop when simulating the polarization device for a 22.5° target ellipticity angle. The results of the simulation are depicted in Fig. 3a. The red line represents the amplitude of the transmitted light after passing through the linear polarizer at different angles, as shown in Fig. 3c, while the blue and green lines correspond to the x and y polarization components of the beam amplitude, respectively. The ellipticity obtained from this simulation is 0.414, and the corresponding ellipticity angle is 22.521 deg according to Eqs. (18–20). Contrary to the traditional approach of designing phase retarders, this method achieves high-precision polarization conversion by aligning the transmitted amplitude component with the target polarization state along each polarization direction, which not only eliminates the search for a target phase difference and maximizes the transmitted amplitude in the traditional metasurface wave plate design process but also improves the accuracy of the polarization transformation. The parameters obtained from the simulation include micro and nanofabrication process parameters, as detailed in the subsequent Materials and Methods section.

Following the manufacturing of the polarization-transformation metasurface, a test experiment is conducted, as depicted in Fig. 3c. The linearly polarized light emitted from the laser passes through a half-wave plate, the polarization-transformation metasurface, a linearly polarized plate, and finally a photodiode. The linear polarizer is affixed to a stepper motor rotation mount (K10CR1/M, Thorlabs) and rotates at a fixed rate to obtain optical power signals with different rotation angles. The optical power test results show that the polarization extinction ratio (PER) is $PER=10{\log }_{10}\frac{0.77}{0.13}(dB)=7.7dB$, represented by the orange line in Fig. 3b. In addition, the calculated ellipticity is 0.407, and the corresponding ellipticity angle is 22.17 deg. Limited by the machining error in the nanofabrication process, this result deviates from the target polarization state by approximately 2%, which satisfies the polarization transformation criteria for elliptically polarized laser-pumped SERF magnetometers.

### Magnetic field measurements

**Fig. 4:**
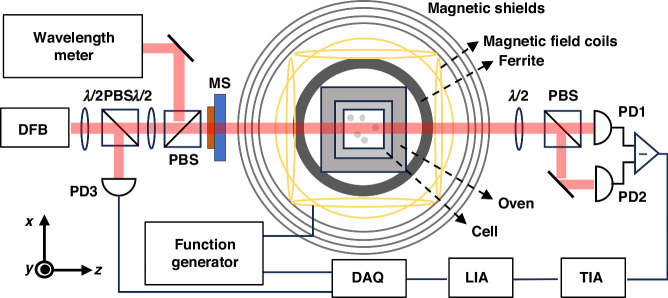
Schematic diagram of the experimental setup for elliptically polarized laser pumped magnetometers. Elliptically polarized light propagating along the z-axis was utilized to polarize Rb atoms and detect the optical rotation angles simultaneously. PBS: polarized beam splitter. TIA Transimpedance amplifier, LIA Lock-in amplifier, DAQ Data acquisition

The magnetic field measurement experiment setup is displayed in Fig. 4. A 3 × 3 × 3 *mm*^*3*^ borosilicate cubic cell with a droplet of ^87^*Rb* metal and 450 Torr of *N*_2_ buffer gas is the key sensing unit of the magnetometer. A boron nitride ceramic oven encased the vapor cell and is heated to 433 K using a twisted pair winding resistor driven by a 240 kHz AC electronic current. The oven temperature is monitored with a nonmagnetic Pt 1000 sensor and controlled by a PID program, which ensures temperature stability with fluctuations of less than 100 mK. For the convenience of the experiment, a commercially available DFB laser is used to emit 795 nm linearly polarized light with 30 GHz detuning. After being transmitted from the metasurface, the circularly polarized component polarizes the alkali-metal atoms, whereas the linearly polarized component is employed to measure the optical rotation angles. The transmitted beam is analyzed using the balanced polarimetry technique, which incorporates a transimpedance amplifier. A multilayer cylindrical magnetic shield composed of three layers of µ-metal and an aluminum shield achieves a quasistatic shielding factor of at least 10^5^, ensuring a low-noise magnetic environment with a residual magnetic field measuring below 5 nT. The outermost layer of aluminum shielding serves to attenuate high-frequency magnetic noise. A cylindrical uniform field coil^35^ is used to manipulate the triaxial magnetic field within the cylindrical magnetic shield.

**Fig. 5:**
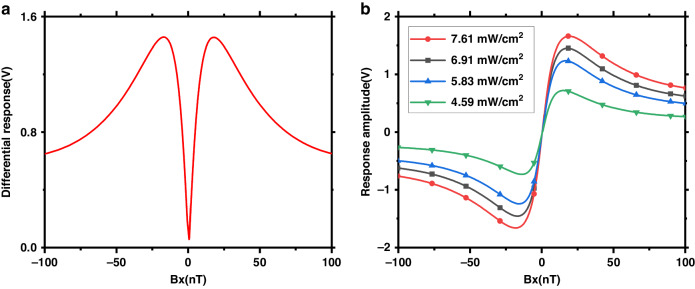
**a** Zero-field resonance as a function of the transverse magnetic field Bx when the By and effective Bz magnetic fields were zeroed. **b** Lock-in amplifier dispersion signal under different incident luminous fluxes

A testing methodology identical to that for a miniaturized elliptical polarization laser pumped magnetometer is used in this study^11,12^. By sweeping a 100 nT DC magnetic field to the x-axis, a narrow zero-field SERF resonance signal is obtained by monitoring the difference in the photodiode signal as a function of the Bx field, as shown in Fig. 5a. Figure 5b shows the lock-in amplifier dispersion signal under different incident luminous fluxes, which can be used for biomagnetism measurements.

**Fig. 6:**
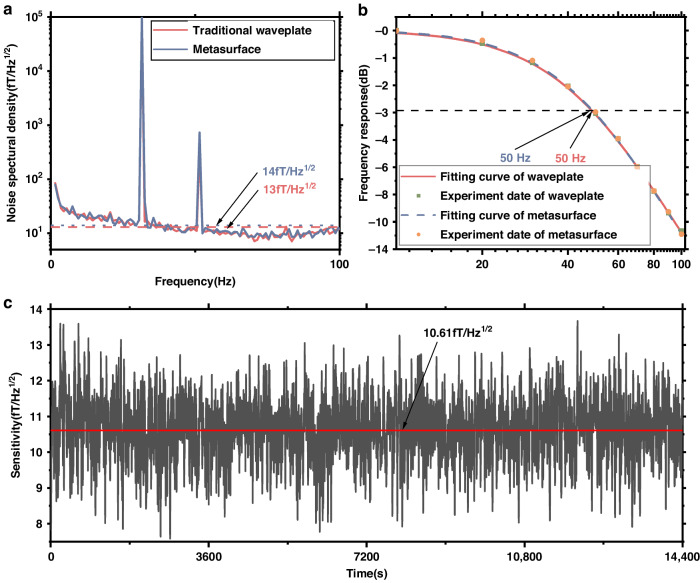
Sensitivity and bandwidth of the magnetometer. **a** The blue and red lines represent the sensitivities of the SERF magnetometer for the traditional waveplate and metasurface, respectively. **b** Frequency response of the 100 pT rms calibrated magnetic field at frequencies of 1–100 Hz and the fitting curve. **c** Four-hour test of the magnetometer sensitivity at 30 Hz. The red line at 10.61 fT/Hz^1/2^ represents the average sensitivity

A 100 pT rms sinusoidal calibration signal is applied at 30 Hz along the z-axis, and the frequency spectrum of the magnetometer response is recorded for 50 seconds. The sensitivity noise floors for magnetometer systems with traditional waveplates and metasurfaces are illustrated by the red and blue lines in Fig. 6a. In Fig. 6b, the normalized frequency response of the magnetometer is presented under the influence of a 100 pT rms calibration magnetic field. The bandwidths at −3 dBm are measured at 50 Hz in both systems. Figure 5c shows the results of a 4 h test of the magnetometer sensitivity, and the red line shows that the 4 h average sensitivity was 10.61fT/Hz^1/2^ @ 30 Hz with the metasurface. The experimental results show that an elliptically polarized laser-pumped SERF magnetometer based on a polarization-transformation metasurface can achieve almost the same high sensitivity as traditional waveplates and is compatible with nanofabrication processes, which is promising for improving the spatial resolution of measurements in MCG and MEG.

The fundamental mechanism of the SERF atomic magnetometer involves laser pumping of alkali-metal atoms with diverse polarization states to influence and observe the quantum states of their outermost electrons. Generally, a larger ellipticity angle of the pumped beam represents a stronger pumping effect, leading to higher atomic polarizability and a higher scale coefficient, while it implies a nonnegligible optical frequency shift, resulting in the generation of a fictitious magnetic field and decreases in the scale coefficient and sensitivity. Therefore, the ellipticity angle is vital to the tradeoff between the fictitious magnetic field and atomic polarizability for compact elliptically polarized laser-pumped SERF magnetometers where alkali atoms are pumped and detected simultaneously by single elliptically polarized light.

In this study, a high-sensitivity (reaching 10.61 fT/Hz^1/2^) elliptical-polarization pumping SERF magnetometer is produced by utilizing a single polarization-transformation metasurface, which is comparable to the current state-of-the-art SERF magnetometer with traditional bulky optical components. The theoretically optimum elliptically polarized beam is designed by combining the metasurface polarization transform method with a computer-assisted optimization algorithm. The polarization-transformation metasurface is fabricated with a 550 nm thick silicon-rich silicon nitride layer on a SiO_2_ substrate and features a 22.17 degree ellipticity angle of transmitted light (less than 2% deviation from the target polarization state) and more than 80% transmittance. This work demonstrates how bulky, conventional optical polarization transform components in compact atomic magnetometers can be fully replaced by metasurfaces based on nanofabrication technology, which has significant potential for exponential volume reduction and resolution enhancement. Furthermore, we explore full-chip integration (including devices such as wave plates, vapor cells, and PBSs) magnetometers for future wearable atomic devices and high-space-resolution biomagnetism imaging.

## Materials and methods

### Optimization algorithm

The algorithm used in this study consists of five main steps^32,33^.

Step 0: Algorithm parameter definition: Parameters (P, w, l, and h) that are associated with the output polarization state are set as variables in the algorithm, and their variation ranges are limited. The initial set of nanopillar particles is created by assigning random values to each particle parameter.

Step 1: Evaluating fitness using a far-field polarization module: Based on the current parameter groups of the nanopillar particles, the FDTD software calculates the ellipticity of the outgoing light of the nanopillar particles as well as the fitness of the parameter groups (ellipticity = short-axis length/long-axis length), and the current parameter groups and the fitness are returned to the algorithm for further computation. The accuracy of the ellipticity calculation will have a direct effect on the optimization process.

Step 2: Updating the optimal value of the optimization parameter: This algorithm compares the ellipticity calculated using the current parameter group to the historical optimal value and, if the calculated value is greater, replaces the historical optimal value with the value calculated with the current parameter group.

Step 3: Updating the speeds and locations of the particles: The parameter set is changed based on the current position and velocity of each particle. The parameter group of the next-generation particles is^34^

21 $$\begin{array}{ll}{v}_{j}^{i+1}=w\cdot {v}_{j}^{i}+{c}_{1}\cdot rand(0,1)\cdot (pbes{t}_{j}^{i}-{x}_{j}^{i})\\\qquad\quad+\,{c}_{2}\cdot rand(0,1)\cdot (gbes{t}^{i}-{x}_{j}^{i})\end{array}$$

where *c*_1_ and *c*_2_ represent the self-learning factor and the group learning factor, respectively, and are generally assigned a value of 2; $rand(0,1)$ represents a random number between 0 and 1; $pbes{t}_{j}^{i}$ and $gbes{t}_{j}^{i}$ represent the optimal position of individual particles and the global optimal position, respectively; and *w* represents the inertia weight, which is expressed as

22 $$w(n)={w}_{\max }-({w}_{\max }-{w}_{\max })\cdot \frac{n}{N}$$

where *N* is the maximum number of iterations and *n* represents the current iteration number.

Step 4: Evaluating the termination conditions: Whether the ellipticity satisfies the specified criteria or the number of iterations has reached the predefined limit is determined. If either of these termination conditions is met, the historical optimal value and parameter group are recorded; otherwise, the next iteration cycle commences.

Following numerous iterations, the algorithm will identify the parameter set that best matches the target ellipticity for the nanopillar.

### Device fabrication

The micro and nanofabrication process begins with a 4-inch glass wafer with a thickness of 500 μm serving as the substrate. A 550 nm thick SiNx layer with a refractive index *n* = 2.74 and extinction coefficient *k* = 0 at 795 nm is deposited onto the substrate to serve as the metasurface material^36^. Subsequently, a 200 nm thick photoresist layer (ZEP520A) is spin-coated onto the SiNx layer for lithography. The E-beam lithography process is employed to generate the pattern of the polarization-transformation metasurface, which is then developed on the photoresist layer. Next, a 50 nm thick Cr layer is deposited onto the SiNx layer to act as a hard mask. Both deposition steps are conducted using electron-beam physical vapor deposition. The structure of the metasurface is subsequently transferred into the Cr hard mask layer by eliminating the residual photoresist through a plasma stripper. Finally, the pattern is transferred into the SiNx layer utilizing an ICP process. The decision to utilize the Cr layer as a hard mask stem from the unavailability of an E-beam photoresist (at a thickness of 200 nm) with sufficient selectivity against SiNx, a requirement for achieving direct ICP at a depth of 550 nm. Furthermore, the thickness of the E-beam resist is constrained by our feature size of 113 nm. Consequently, a lift-off process involving a hard mask is indispensable for the fabrication of this polarization-transformation metasurface.
